# 0D-1D Hybrid Silicon Nanocomposite as Lithium-Ion Batteries Anodes

**DOI:** 10.3390/nano10030515

**Published:** 2020-03-12

**Authors:** Sergio Pinilla, Sang-Hoon Park, Kenneth Fontanez, Francisco Márquez, Valeria Nicolosi, Carmen Morant

**Affiliations:** 1 Department of Applied Physics, Laboratory of Coatings and Nanostructures and Instituto Nicolás Cabrera, Universidad Autónoma de Madrid (UAM), Cantoblanco, 28049 Madrid, Spain; PINILLAS@tcd.ie; 2 School of Chemistry, CRANN & AMBER, Trinity College Dublin, 02 Dublin, Ireland; marka0819@hanmail.net; 3 Nanomaterials Research Group, Department of Chemistry, Universidad Ana G. Méndez-Gurabo Campus, 189 St Rd km 3.3, Gurabo, PR 00778, USA; kfontanez12@email.uagm.edu (K.F.); fmarquez@suagm.edu (F.M.)

**Keywords:** silicon, lithium ion batteries, nanomaterials, 0D, 1D

## Abstract

Lithium ion batteries (LIBs) are the enabling technology for many of the societal changes that are expected to happen in the following years. Among all the challenges for which LIBs are the key, vehicle electrification is one of the most crucial. Current battery materials cannot provide the required power densities for such applications and therefore, it makes necessary to develop new materials. Silicon is one of the proposed as next generation battery materials, but still there are challenges to overcome. Poor capacity retention is one of those drawbacks, and because it is tightly related with its high capacity, it is a problem rather difficult to address with common and scalable fabrication processes. Here we show that combining 0D and 1D silicon nanostructures, high capacity and stability can be achieved even using standard electrode fabrication processes. Capacities as high as 1200 mAh/g for more than 500 cycles at high current densities (2 A/g) were achieved with the produced hybrid 0D/1D electrodes. In this research, it was shown that while 0D nanostructures provide good strain relaxation capabilities, 1D nanomaterials contribute with enhanced cohesion and conductive matrix integrity.

## 1. Introduction

Li-ion batteries (LIBs) have become inseparable companions of the advances that society has experienced in the last 15 years. They are a crucial part of the vast majority of portable electronics, and as new applications emerge, greater energy storage capabilities are required. Specially, the need to develop electrical vehicles (EVs) with performance and autonomy similar to those of internal combustion engines (ICEs), is a challenge that goes through new developments in LIBs.

Current technology based on graphite anodes, with a capacity of 372 mAh/g, fail to deliver the needed gravimetric capacity of 1000 mAh/g that has been proposed as optimal to meet future demands [1,2]. This, combined with the incompatibility of graphite with some of the common electrolyte solvents [3,4], makes necessary the use of other high capacity and low voltage anode materials. In this scenario, silicon stands as one of the most outstanding options, with a theoretical capacity close to 3590 mAh/g and a great availability, it has been predicted to be incorporated in commercial batteries between 2025–2030 [5].

However, the high specific capacity of Si as anode material in LIBs is counterbalanced by huge volume variations during the Li-Si alloying/dealloying processes [6,7]. This expansion and contraction has as side effects a large irreversible loss of capacity due to the continuous SEI formation [8] and a poor capacity retention caused by the pulverization of the silicon [9]. To avoid these drawbacks, the nanostructuring of silicon has been proposed as a promising strategy for anodes fabrication [10]. Taking advantage of the facile strain relaxation that the nanometric size provides to the structures [11,12] and the improved diffusion achieved through a higher surface to volume ratio [13], nanomaterials are capable of effectively mitigate the pulverization and continuous SEI formation while allowing higher charge/discharge rates.

However, Si nanomaterials still face significant challenges. Their very high surface area produces large amounts of SEI that is renewed upon cycling [4], and their shrinkage after delithiation tends to isolate material in each cycle [14]. To address these issues, different strategies have been explored: (i) A carbon coating on the Si surface. Carbon allows the diffusion of $Li^{+}$ to the underlying Si, electrically connects the particles, and an homogeneous coverage can minimize the SEI formation [15]. This approach is easy to implement but, usually, the coating cannot withstand the expansion of Si volume and ends up breaking. Some authors have been able to solve this problem [16] but employing a significantly more complex approach which is not easy to export to high mass production. (ii) Mixing the Si with C-based nanomaterials. Carbon nanomaterials have similar advantages to those of the amorphous carbon discussed above, and can more easily adapt to the volume changes of silicon [17,18]. However, they do not solve the SEI formation problem, and, despite reduced, the isolation of Si particles can still occur. (iii) New polymer binders and chemical bonding to them. The performance of binders in Si anodes depends largely on particle size and morphology [19], therefore, it is necessary to adapt them to the specificities of the Si material used. Furthermore, it has been proven that the functionalization of Si to link it to the binder, also helps improve cycling stability [20]. Despite being promising and easy to implement, this last approach still needs a significant break through to catch up with the other strategies.

In this work, a different approach is proposed. We studied the use of different lengthen Si nanomaterials (0D and 1D) to create an interconnected network that minimizes particle isolation. The research was started studying the characteristics of several Si nanostructures in order to select the most promising materials. We used a range of commercially available Si nanomaterials as well as an in-house synthesized Si material produced by an easy and scalable technique [21]. It was found that, from the materials analysed, the smallest commercial nanoparticles had the highest overall performance while the 1D synthesized material showed improved network integrity. The combination of these small 0D and long but thin 1D Si nanomaterials, resulted in a performance of 1200 mAh/g that was stable even after 500 cycles. The developed hybrid electrode, based on commercial nanoparticles and in-house synthesized nanowires, meets the optimum anode requirements, with a processing technique compatible with current battery technology. Also, its performance, in terms of long cycling stability, is among the highest reported up to date for silicon based-anodes.

## 2. Materials and Methods

### 2.1. Synthesis of Silicon Nanowires

Silicon nanowires (SiNWs) were produced by a wet etching method known as MACE (Metal Assisted Chemical Etching) [22,23]. Monocrystalline 3-inch Si wafers, p-type with <100> orientation (1–10 Ωcm, boron-doped, single side polished, 375±25μm thick, PI-KEM) were used as substrates. The standard two-step MACE procedure, described in detail in our previous works [24,25], was followed. The whole Si wafer was cleaned by consecutive sonication in acetone, isopropyl alcohol and DI-H${}_{2}$O, for 10 min each. Subsequently, it was immersed in a piranha solution (18 M H${}_{2}$SO${}_{4}$: 10 M H${}_{2}$O${}_{2}$, 3:1 *v*/*v*) for 30 min and rinsed thoroughly with DI-H${}_{2}$O. Then, the silicon dioxide formed on the surface was removed by HF (5%) treatment for 5 min. After this cleaning, the MACE was performed on small pieces of the Si wafer. Si fragments were immersed in a AgNO${}_{3}$/HF bath (0.01 M $AgNO_{3}$ and 4.8 M HF) for a period of 2 min, obtaining a uniform coverage of Ag nanoparticles on the silicon surface. Afterwards, it was immersed in a second HF/H${}_{2}$O${}_{2}$ bath (molar ratio HF/H${}_{2}$O${}_{2}=24$) for 440 min. During this period, a longitudinal etch was produced along the entire thickness of the sample. Following this procedure for a sufficient etching time, the total thickness of the silicon sample was transformed into SiNWs.

Once the process was completed, the sample was rinsed in DI-H${}_{2}$O for cleaning. The SiNW arrays were separated from the mother Si wafer by ultrasonication for 2 min in Isopropyl alcohol (IPA, Aldrich). After this sonication, there was no solid macroscopic remains of the original sample and all the nanowires were dispersed in IPA. Finally, the solutions were centrifuged at low speed (300 rpms) to separate the nanowires from the micrometric pieces of un-etched material.

### 2.2. Electrodes Preparation, Battery Assembly and Electrochemical Characterization

**Figure 1:**
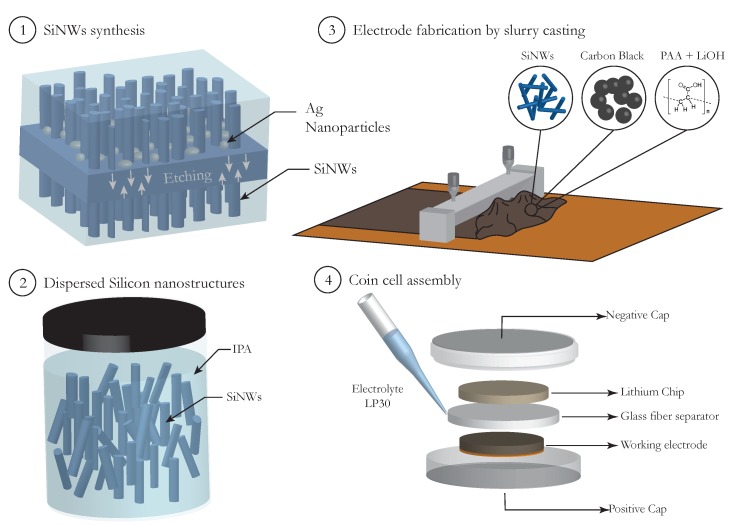
Scheme of the different steps (1–4) for the preparation of batteries with MACE SiNWs electrodes.

The fabrication of electrodes was conducted by the mixture of the active material with binders and conductive additives. The active material was constituted by Si nanostructures, i.e., Si nanowires or Si nanoparticles, or composites with both nanostructures. All the electrodes in this work were prepared following the same recipe, which is described in the subsequent lines. The working electrodes were prepared with 70% of Si material, 20% of Carbon Black (Timical Super C65, MTI Corp) and 10% of Li polyacrylate (Li-PAA) (3 g PAA (35%) + 0.6 g LiOH + 78.9 g DI-H${}_{2}$O, prepared as described by Eberman et al. [26]). The mixing was accomplished by manual milling and, subsequently, the slurry was spread on a copper foil (MTI Corporation 99.99%, 9 μm thick) by using a doctor blade at a height of 150 μm. Then, it was dried in a vacuum oven at 40 ${}^{{}^{\circ}}$C for 5 h to avoid the formation of cracks [27]. The mass loadings achieved ranged between 1.3–2.0 mg/cm${}^{2}$. Once dried, the resulting electrodes were cut to the desired round disc (12 mm diameter) with a electrode puncher (EL-Cut, provided by EL-CELL). Afterwards, CR2032-coin cells were assembled in half-cell configuration with a borosilicate glass fiber separator (Whatman GF/B) and an electrolyte composed by 1 M LiPF${}_{6}$ in ethylene carbonate and dimethyl carbonate (EC:DMC, 1:1 *v*/*v*, supplied by Sigma-Aldrich). Once assembled, the batteries were allowed to homogenize for at least 4 h before being measured. A schematic of the whole fabrication process is displayed in Figure 1. The electrochemical characterization was performed with a 12-channel Arbin Instruments BT2143 workstation, at room temperature (25 ${}^{{}^{\circ}}$C). Galvanostatic charge/discharge tests were conducted in a voltage range of 0.01–1.2 V vs. Li${}^{+}$/Li, at different current densities. The cyclability of the electrodes was evaluated at 2 A/g (∼1/2 C) after initial formation cycle at 0.5 A/g (∼1/8 C). The discharge rate-capabilities of the electrodes were investigated using symmetrical charge/discharge conditions with rates ranging from 1/20 C to 1.5 C.

### 2.3. Material Characterization

Scanning Electron Microscopy (SEM) images of the Si nanostructures, as well as the prepared electrodes, were obtained with a Zeiss Ultra Plus (Carl Zeiss, Germany) at an acceleration voltage of 5 keV. X-Ray Diffraction was performed with a Bruker D5000 powder diffractometer equipped with a monochromatic Cu Kα radiation source. XRD patterns were collected between $5^{{}^{\circ}}<\theta <90^{{}^{\circ}}$, with a step size of 2θ = 0.02 and a time per step of 163 s.

## 3. Results and Discussion

### 3.1. Morphology of the Nanomaterials

In the first instance, SiNWs were fabricated by MACE and used as anode material in LIBs. Subsequently, for comparison, two commercially available Si-based nanomaterials were also studied as battery anodes: commercial silicon nanoparticles (SiNPs, Alpha Aesar) and commercial SiNWs/SiNPs mixture (US Research Nanomaterials).

**Figure 2:**
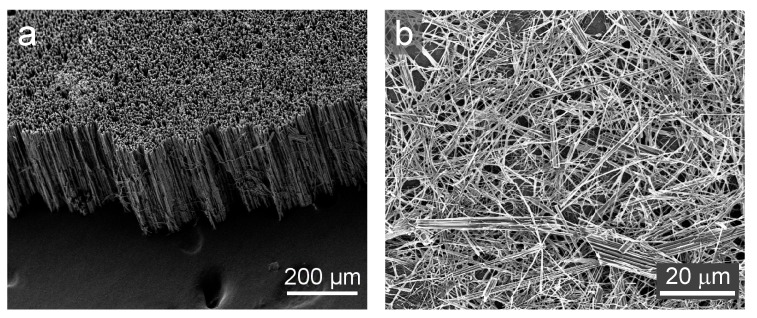
(**a**) Silicon wafer after the completion of the MACE, (**b**) Silicon nanowires deposited from IPA dispersion.

As described in the experimental section, SiNWs were obtained by etching a full wafer, Figure 2a. The total thickness of the wafer was completely engraved, generating SiNW arrays vertically aligned along the normal axis of the wafer. The average height of the etched wafer was 240 μm. It is worth mentioning that this value is lower than the initial thickness of the wafer. This has been previously reported and is due to the dilution of the nanowires tips after a prolonged exposure to the enchants. Despite that the tips of the nanowires are free from the presence of catalyst (silver nanoparticles in this case), a slow etching can still happen at the bare silicon [24,25]. After sonication for 2 min in IPA and low speed centrifugation, SiNWs dispersion was achieved. During sonication and centrifugation processes, SiNWs are almost completely separated and shortened to lengths in a wide range, 1–50 μm, as is observed in Figure 2b.

**Figure 3:**
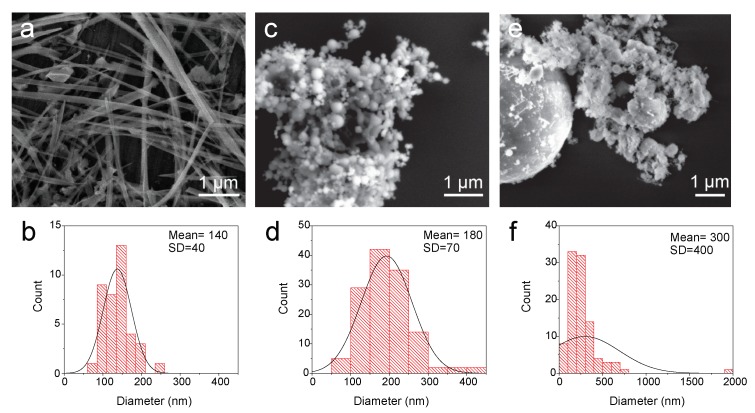
SEM images of MACE SiNWs (**a**), SiNPs from Alpha Aesar (**c**) and SiNWS/SiNPs from US nano (**e**). (**b**,**d**,**f**) are the histograms of above-mentioned samples.

For comparison, the commercial silicon nanomaterials were selected so their diameters were similar to the MACE silicon nanowires. This consideration is very important because the stability of silicon materials upon lithiation is highly dependent on the particles size [12,28], therefore only equally lateral sized materials makes a fair comparison. Figure 3 shows representative SEM images of the Si nanostructures used for the first stage of the current study, namely MACE-SiNWs (Figure 3a), SiNPs-AlphaAesar (Figure 3b) and SiNWs/SiNPs-US (Figure 3c). The corresponding histograms indicate that the diameters of the fabricated SiNWs-MACE are very homogenous, 140±40 nm, while the commercial SiNPs slightly increase their average diameter, 180±70 nm. On the other hand, the mixture of SiNWs/SiNPs provided by US research nanomaterials, presents a wide range of dimensions. The small wires appear aggregated to nano- and micro-sized particles (Figure 3c); the resulting statistical analysis of the particulate sizes (histogram in Figure 3f) shows a dispersion value larger than the mean value, 300±400 nm. Supporting Information (Figure S1) includes general views of this heterogeneous material, where the presence of big structures is evident. The sizes dispersion of the commercial mixture may have an impact on the LIB performance, limiting the life of the cell and its rate.

### 3.2. Electrochemical Characterization of SiNWs and Comparison with Commercial Materials

**Figure 4:**
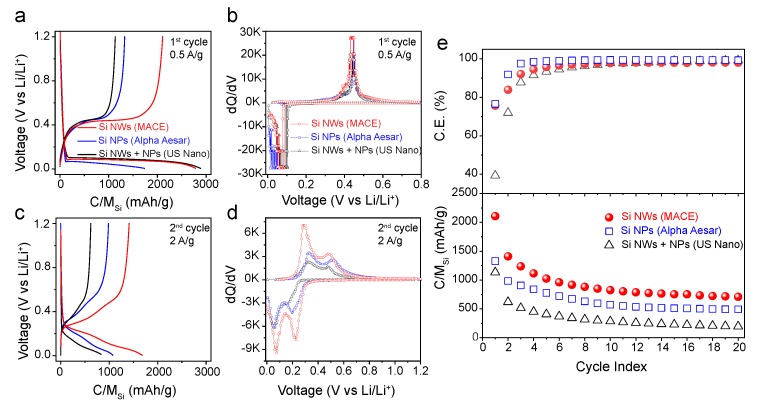
Electrochemical performance of selected material, as labelled (MACE-SiNWs, SiNPs- AlphaAesar, SiNWs/SiNPs-US). (**a**,**c**) are galvanostatic charge-discharge (GCD) curves of the three selected materials during the 1st (≈1/8 C) and 2nd cycles (≈1/2 C) of the galvanostatic cycling, respectively. (**b**,**d**) are the dQ/dV profiles obtained from the GCD curves corresponding to each electrodes. Figure (**e**) shows the coulombic efficiency and specific capacity of the selected materials.

In the next step, all the above Si nanostructures were evaluated as anodes for LIBs. Galvanostatic Charge-Discharge (GCD) curves, between 0.01–1.2 V are shown in Figure 4a (first cycle) and Figure 4c (second cycle). The resulting differential capacity (dQ/dV) curves, extracted from the GCD profiles, are presented in Figure 4b (first cycle) and Figure 4d (second cycle).

All these Si-based electrodes show the characteristic cathodic peak lower than 0.15 V in the dQ/dV curves for the first lithiation (Figure 4b). The mentioned peak is associated by a constant plateau in the GCD profiles (Figure 4a), and corresponds to the Li-alloying of crystalline Si to form amorphous Li${}_{x}$Si [29,30] by the reaction:

(1) $$\mathrm{c-Si}+x\mathrm{Li}\to {\mathrm{a-Li}}_{x}\mathrm{Si}$$

In this first lithiation, the change of slope at voltages < 50 mV indicates another two-phase transition from amorphous lithiated silicon to crystalline Li${}_{15}$Si${}_{4}$ [31]. Likewise, during the first delithiation, all the electrodes show an anodic peak at ca. 0.45 V in the dQ/dV curves (Figure 4b), which is associated with the corresponding plateau in GCD profiles (Figure 4a). This peak is assumed to correspond to the two-phase reaction from the delithiation of the crystalline Li${}_{15}$Si${}_{4}$ to form a-Si [29,30,31].

(2) $${\mathrm{Li}}_{15}{\mathrm{Si}}_{4}\to 4\mathrm{Si}+15\mathrm{Li}$$

During the second cycle (Figure 4c,d), all the materials exhibit the characteristics of amorphous Si [26,32,33], composed by two broad peaks in each lithiation/delithiation process. The lack of two-phase transition peaks indicates that the Si-Li richest specie is the amorphous Li${}_{15}$Si${}_{4}$ [30], giving a theoretical nominal capacity of 3578 mAh/g. The exact compounds formed in each reaction are unknown, but it is well established that the lithiation is produced by two consecutive incorporations of Li into amorphous phases of Si-Li [33].

From Figure 4a,e, it can be observed that MACE-SiNWs presents the highest charge capacity among the analysed electrodes. Even though the first lithiation capacities both for the MACE-SiNWs and commercial SiNWs/SiNPs-US electrodes are similar (Figure 4a), the subsequent charge indicates that the MACE-SiNWs electrode retained double delithiation capacity. As a result, the initial coulombic efficiency of the MACE electrode (75%) is higher than the one for commercial SiNWs/NPs-US (38%) (Figure 4e). In consecutive cycles (Figure 4e), the capacity of all electrodes gradually decays, showing a capacity retention of 34% (MACE-SiNWs), 37% (SiNPs-AA) and 17% (commercial SiNWs/NPs-US) after 20 cycles. This effect is common in Si-based electrodes and is generally caused by pulverization and loss of electrical contact [34,35]. Nevertheless, the MACE-SiNWs electrode displays better performance compared to the other commercial Si-based electrodes, which retain less than 500 mAh/g after 20 cycles compared to the 710 mAh/g of the MACE-SiNWs (Figure 4e). The substantial capacity difference between the MACE-SiNWs electrodes and the other Si nanostructures, might be ascribed to the longitudinal dimension of the nanowires. Their length (up to 50 μm) makes them less susceptible to be isolated from the network and therefore more of the Si mass is electrochemically active. Moreover, the lower surface area in comparison with the other 1D nanomaterias analysed (the commercial SiNWs/NPs-US), can also contribute to better performance by reducing capacity loss due to SEI formation (as shown in Figure 4e by a higher C.E.).

dQ/dV curves during the first lithiation/delithiation cycle (Figure 4b) provide further evidence of the advantage for the MACE-SiNWs electrode. As mentioned, all of these Si-based electrodes show the characteristic sharp cathodic and anodic peaks; however, the voltage positions in which they appear are not the same. The anodic peak (de-alloying of Li-Si phase) for the MACE-SiNWs electrode initially occurs at a lower potential, indicating that a relatively low anodic overpotential was induced for the MACE-SiNWs electrode during delithiation. In the second cycle, Figure 4d, this effect is repeated, and the MACE-SiNPs show again a substantially lower anodic potential than the other two silicon materials. An overpotential usually indicates a higher internal resistance in the electrode that shifts the reaction potentials due to polarization, and degrades the performance of the cell. This resistance may come from a poor electrical contact between the active material and the conductive matrix [16], or from the diffusion kinetics in the material [36]. Since all our materials have the same composition and roughly same diameters, it can be deducted that the differences between overpotentials mainly come from the contact with the conductive matrix, being the MACE-SiNWs those that stand out over the commercial materials. Please note that what we measure in our configuration is not the overpotential but the overvoltage. However, since lithium is considered to have a zero polarization voltage, effectively these two magnitudes are the same.

### 3.3. Hybrid Electrode Fabrication and Performance

The results described above prove that, despite having lower surface to volume ratio than commercial particles, MACE-SiNWs display better electrochemical performance. In order to deepen into this matter, we added to our study substantially smaller particles, 100 nm sized nanoparticles (from US Research Nanomaterials).

**Figure 5:**
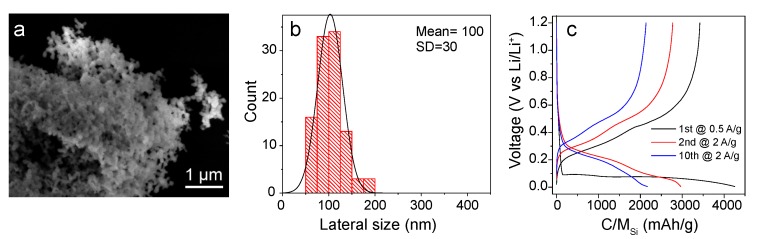
Representative SEM image of the SiNPs from US Research Nanomaterials (**a**). Size distribution of the SiNPs (**b**). GCD curves of the first 10 cycles of the SiNPs LIBs (**c**).

SEM images (Figure 5a,b) of the particles, namely SiNPs-US, confirm an homogeneous size of (100±30 nm). The electrode fabrication process and electrochemical characterization were followed as for previous materials, giving the results shown in the GCD curves displayed in Figure 5c. From the GCD, it can be appreciated an outstanding electrochemical performance of the electrode after 10 cycles, maintaining a specific capacity around 2100 mAh/g. In comparison, MACE-SiNWs electrodes (Figure 4a) only retained ca. 820 mAh/g after the same number of cycles. Interestingly, the GCD profiles do not show the sharp slope change associated to the two-phase transition peak above discussed, corresponding to the delithiation of the crystalline Li${}_{15}$Si${}_{4}$. This phase transition cannot be observed even for the first cycle, which contrasts with all the tested Si nanomaterials with bigger size. This might be explained by the lack of crystallinity in the SiNPs-US structure. However, XRD spectra of the four different materials (Figure S2) do not show any substantial difference. We can therefore evict that all four materials have similar crystallinity. The only plausible explanation is that the formation of the crystalline Li${}_{15}$Si${}_{4}$ phase, only occurs in particles sizes above a threshold (slightly over 100 nm), and is associated with the cracking of the particles [12,32]. As can be seen from the difference of performance between the different sized materials, this effect can have a severe influence on the overall device performance.

**Figure 6:**
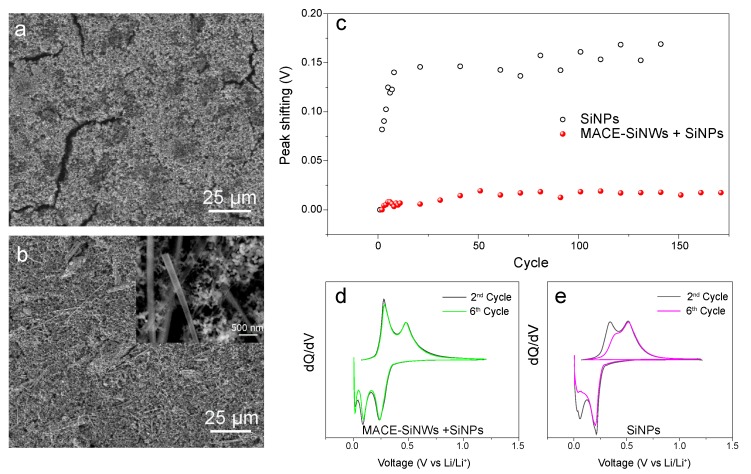
SEM images of a SiNPs-US electrode, (**a**), and the hybrid MACE-SiNWs/SiNPs-US electrode, (**b**). Inset in (**b**) is displayed a closer SEM image of the SiNPs/SiNWs mixture. In (**c**), is represented the voltage shifting of the delithiation peaks of the SiNPs and hybrid electrodes along 150 cycles. The peaks position were obtained from the dQ/dV curves such as those shown in (**d**,**e**).

On the other hand, the GCD curves of the SiNPs-US electrodes (Figure 5c) reveal a significant shift of the delithiation voltages as the cycles progress. This increased overpotential indicates a degradation of the contact with conductive matrix, that, as was previously reported, hinders the cycling performance of the battery in the long term. This effect was investigated more in depth, paying attention to the morphology of the electrodes. SEM images of SiNPs-US electrodes (Figure 6a) revealed that even before cycling, the electrodes presented huge cracks throughout the surface. This is symptomatic of poor cohesion, which could explain the overpotential observed in the GCD curves.

In order to take advantage of the high electrochemical capacity of the commercial SiNPs-US, while keeping the good integrity (and low overpotential) of the electrical network formed by the MACE-SiNWs, it was decided to study hybrid electrodes, made of the two combined systems. Electrodes made of the 0D-1D hybrids were fabricated by mixing SiNPs-US and MACE-SiNWs in a 1:1 ratio. Firstly, it was observed that the presence of MACE-SiNWs in the hybrid provided structural integrity to the electrode, avoiding the cracking previously seen in electrodes solely made of SiNPs-US material (Figure 6b). Higher magnification images (inset of Figure 6b) of the hybrid revealed a homogeneous distribution of both MACE-Si-NWs and SiNPs-US in the electrode. The SiNWs were found surrounded by the NPs, preventing the formation of separated clusters of SiNWs or SiNPs.

In order to further investigate the degradation of the electrodes during cycling, the dQ/dV curves of the hybrid and SiNPs electrodes were obtained and analyzed (Figure 6d,e respectively). For both electrodes, the shift of the first delithiation peak was tracked along 150 cycles and represented in Figure 6c. In this graph, it can be clearly seen that during the first 15 cycles the SiNPs sample shows a pronounced increase of the overpotential, being especially sharp between the two first cycles, probably due to the rearrangement of the network caused by the volume expansion and SEI formation. On the other hand, the hybrid electrode shows a much lower overpotential even during the first cycles. Definitively, the absence of overpotential in the hybrid electrode highlights a better integrity of the composite, which contributes to improved the capacity retention.

**Figure 7:**
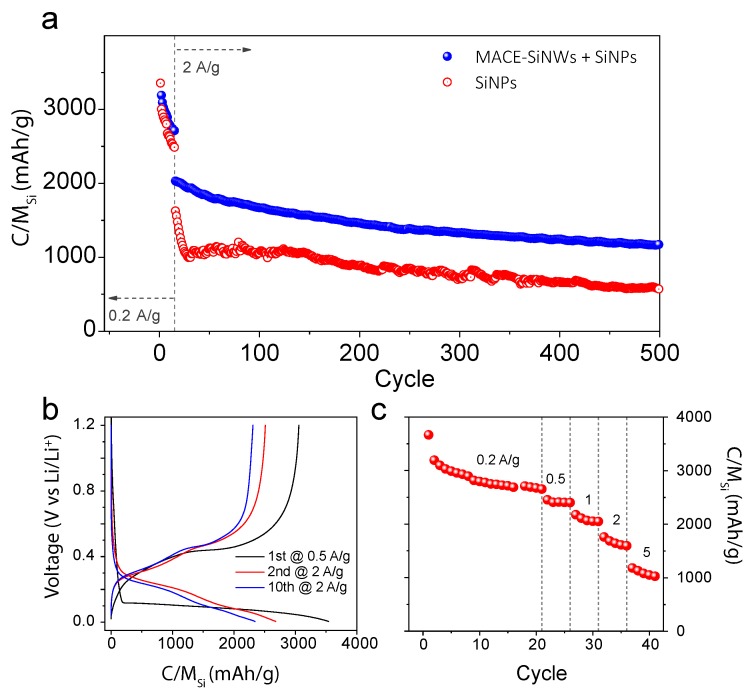
(**a**) Comparison between the cycling performance of the hybrid MACE-SiNWs/SiNPs and the SiNPs-US electrodes. (**b**) GCD curves of the hybrid electrode, and (**c**) shows the rate performance of the hybrid electrode.

Additionally, the electrochemical characterization of the hybrid electrode (Figure 7b) revealed a first delithiation capacity of 3050 mAh/g, which is between the capacity values for single component Si-based electrodes (3400 mAh/g for the SiNPs electrode and 2100 for the MACE SiNWs electrode). This is very common feature observed for the general mixture system under lever rule, but surprisingly, this hybrid electrode maintained a much higher specific capacity in further cycling. After 10 cycles, the delithiation capacity for the hybrid electrode (2307 mAh/g, Figure 7b) is higher than for the electrode made of SiNPs alone (2135 mAh/g, Figure 5c) despite of its lower specific capacity values during initial cycling stages.

To examine the long-term cycling stability of these Si-based electrodes, charge and discharge measurements were carried out at a constant current density of 2 A/g for 500 cycles, as shown in Figure 7a. While the specific capacity of SiNPs-US electrode is markedly reduced to ca. 1000 mAh/g at the earlier cycling stage (after 50 cycles), the MACE-SiNWs/SiNPs-US hybrid electrode only shows a gradual decay of its capacity throughout the whole cycling. After 500 cycles, the hybrid electrode retained a specific capacity of almost 1200 mAh/g, more than twice that of SiNPs-US (580 mAh/g). The capacity displayed by the hybrid electrode after 500 cycles, is not only impressive when compared to the starting material, but it is also positioned in the high end of performance among the state-of-the-art nano-Silicon based anodes (as shown in Table 1). Additionally, the hybrid electrode exhibits quite competitive rate performance when cycled at current densities from 0.2 to 5 A/g (Figure 7c). The hybrid electrode only suffered a 44% decrease in capacity when the current density was increase by a factor of 10 (0.5–5.0 A/g) outperforming, in this aspect, other state-of-the-art silicon composites [37,38,39].

The results presented above demonstrate the exceptional energy storage properties of the hybrid electrode fabricated with long and thin MACE-SiNWs and extra small SiNPs. This is probably due to the synergistic effect of the different dimensional structure (0D-1D hybrid) that combines the stable expansion capabilities of the SiNPs and the good electrode integrity of the SiNWs. Its performance has proven to be superior to any commercial Si-based electrodes tested, to the components separately and it is highly positioned among the state-of-the-art nanoSi-based anodes.

## 4. Conclusions

To summarize, we have explored an easy route to improve the long cycling stability of silicon anodes by combining 0D and 1D Si nanomaterials. In the first stage of the work, the electrochemical performance of a wide range of Si nanomaterials with similar lateral sizes were studied, showing that the MACE synthesized SiNWs, due to their high aspect ratio, are less susceptible to loss of contact and therefore, retain higher capacity after initial cycles. This very interesting property was further exploited by combining the SiNWs with smaller SiNPs that display much better capacity but serious degradation issues. The hybrid 0D/1D electrodes outperformed the single component electrodes, maintaining a capacity of 1200 mAh/g even after 500 cycles. This extraordinary performance was compared with other state-of-the-art Si-based electrodes, being among the highest reported stable capacities in nano-silicon anodes. The composite stability, even after long cycling, and the simple approach for the electrode fabrication, which is based on the industry standards, make this hybrid nanocomposite a very attractive battery material that would meet the performance demands of the next generation LIBs.

## Figures and Tables

**Table 1:** Key parameters and electrochemical performance of our 0D-1D hybrid composite and several nanoSi-based state-of-the-art anodes reported in the literature. Only references showing long cycling stability (>200 cycles) were included in this table.

Si Material	Fabrication Method	Electrolyte	Electrochemical Performance	Ref
SiNWs/SiNPs	Slurry (7:2:1)	EC/DMC	1200 mAh/g, 500 cycles, 2 A/g	This work
SiNPs@C	Slurry (6:2:2)	EC/DEC + 5% FEC	1279 mAh/g, 500 cycles, 2 A/g	[37]
SiNPs@Cellulose nanofibers	Slurry (70:15:15)	EC/DEC + 10% FEC	808 mAh/g, 500 cycles, 2 A/g	[38]
SiNPs@C	Slurry (8:1:1)	EC/DEC + 1% VC	≈1300 mAh/g, 500 cycles, 1/2 C	[16]
SiNPs@r-GO	Slurry (70:15:15)	EC/DMC	≈950 mAh/g, 250 cycles, 1 A/g	[39]
DCS-SiNPs	Slurry (8:1:1)	EC/PC	≈1500 mAh/g, 500 cycles, 1/5 C	[17]
MCS-SiNPs	Slurry (6:2:2)	EC/EMC + 5% FEC	1070 mAh/g, 500 cycles, 1/5 C	[40]
GO-SiNPs paper	Free standing (40% Si)	EC/DMC	≈750 mAh/g, 500 cycles, 0.4 A/g	[41]
SiNPs:H@PAA	Slurry (6:2:2)	EC/DEC/DMC	≈1700 mAh/g, 300 cycles, 0.34 A/g	[20]

## Supplementary Materials

The following are available online at https://www.mdpi.com/2079-4991/10/3/515/s1, Figure S1: SEM images of commercial SiNWs/SiNPs-US, Figure S2: XRD spectra of the silicon nanomaterials studied.

## Abbreviations

The following abbreviations are used in this manuscript:

EVs	Electric Vehicles
ICEs	Internal Combustion Engines
LIBs	Lithium Ion Batteries
SiNWs	Silicon Nanowires
SiNPs	Silicon Nanoparticles
MACE	Metal Assisted Chemical Etching
SEM	Scanning Electron Microscope
XRD	X-ray Diffraction
GCD	Galvanostatic Charge-Discharge
dQ/dV	Differential Capacity
