# Supplementary material for: 0D-1D Hybrid Silicon Nanocomposite as Lithium-Ion Batteries Anodes
Source: Nanomaterials (Basel). 2020 Mar 12;10(3):515. doi: 10.3390/nano10030515 (PMC7153466; doi:10.3390/nano10030515)
Supplement: Supplementary file 1 [file nanomaterials-10-00515-s001.zip › 5.- Supporting Information/Supporting Information.pdf]

Supplementary Materials: 0D-1D Hybrid Silicon Nanocomposite as Lithium-Ion Batteries anodes

Sergio Pinilla [1,2,†], Sang-Hoon Park [2], Kenneth Fontanez [3], Francisco Márquez [3], Valeria Nicolosi [2*] and Carmen Morant [1*]

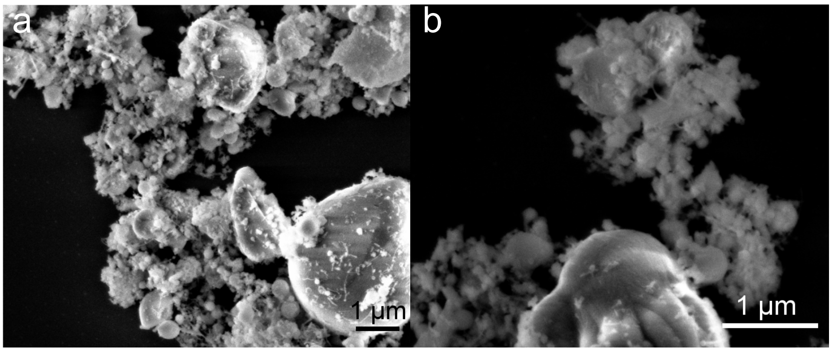

Figure S1. SEM images of SiNWs/SiNPs-US nanomaterial. Different areas are displayed, a) and b), showing non homogeneous particle size.

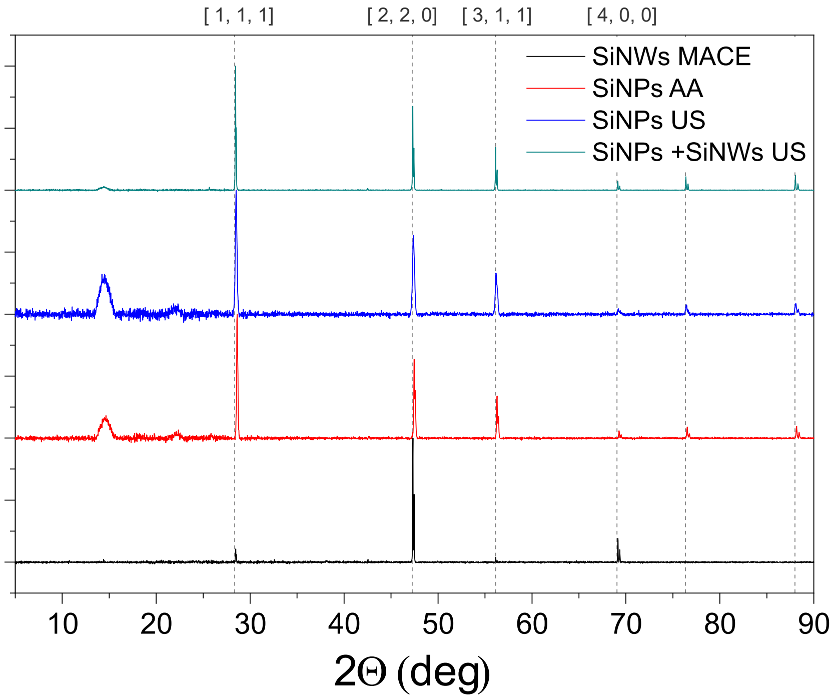

Figure S2. XRD patterns of the four studied Si nanomaterials. The dashed lines indicates Si peak positions of different crystalline orientations.
