# Supplementary figures and images for: 0D-1D Hybrid Silicon Nanocomposite as Lithium-Ion Batteries Anodes
Source: Nanomaterials (Basel). 2020 Mar 12;10(3):515. doi: 10.3390/nano10030515 (PMC7153466; doi:10.3390/nano10030515)

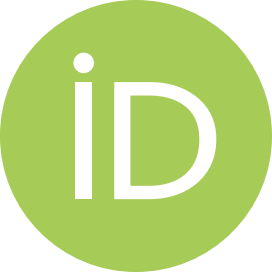

Supplement: Supplementary file 1 [file nanomaterials-10-00515-s001.zip › 5.- Supporting Information/Definitions/logo-orcid-eps-converted-to.pdf]

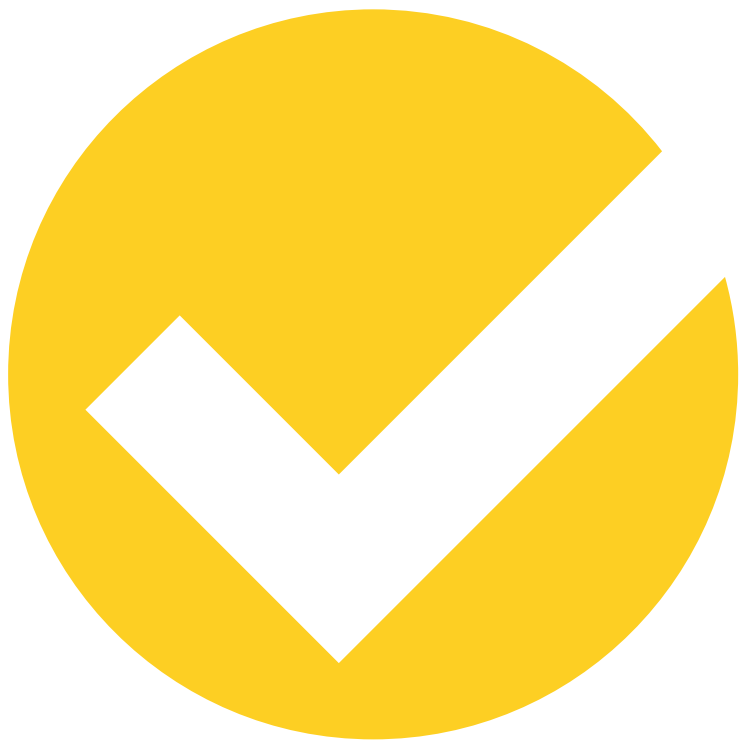

check for  
updates

Supplement: Supplementary file 1 [file nanomaterials-10-00515-s001.zip › 5.- Supporting Information/Definitions/logo-updates.pdf]
